# Supplementary figures and images for: The epididymis contributes to sperm DNA integrity and early embryo development through Cysteine-Rich Secretory Proteins
Source: eLife. 2025 Apr 28;13:RP97105. doi: 10.7554/eLife.97105 (PMC12037180; doi:10.7554/eLife.97105)

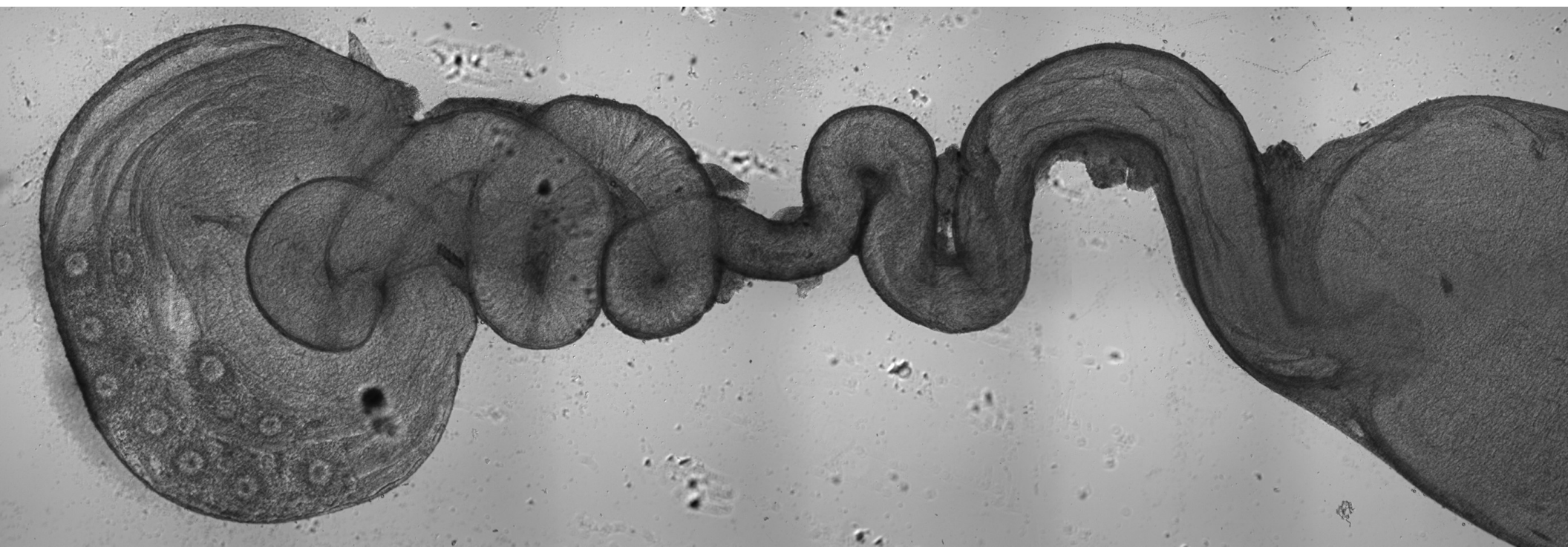

Supplement: Figure 1—source data 1. [file elife-97105-fig1-data1.pdf]

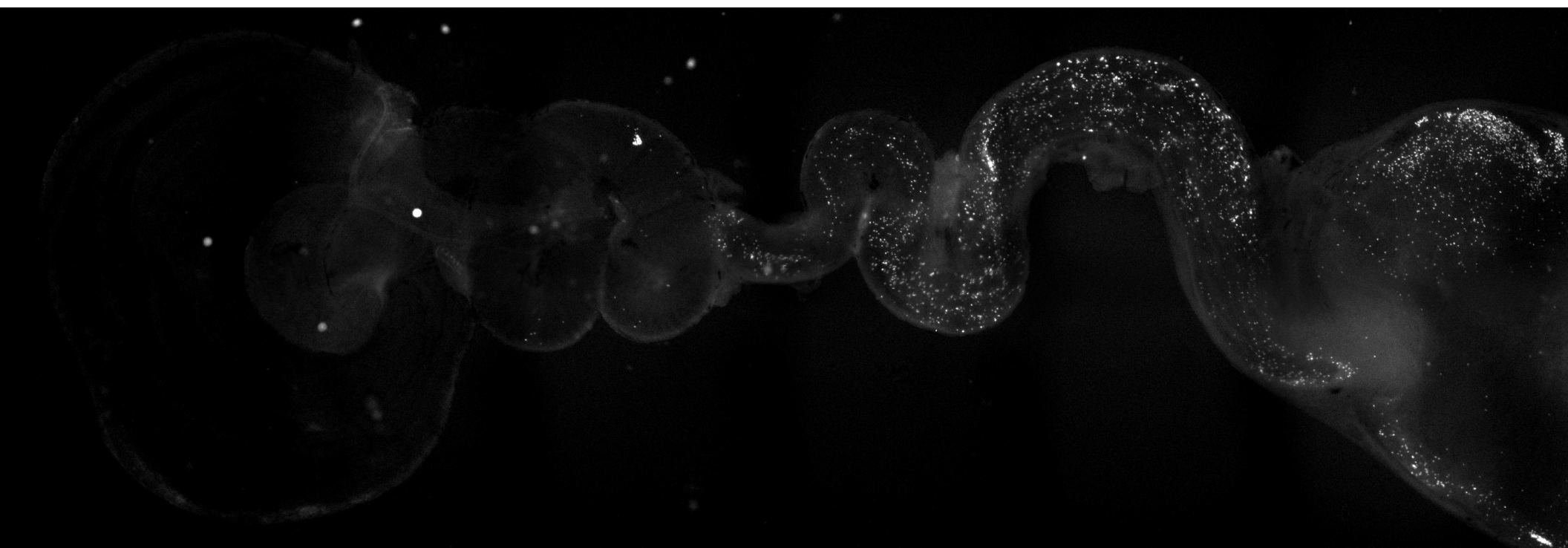

Supplement: Figure 1—source data 2. [file elife-97105-fig1-data2.pdf]

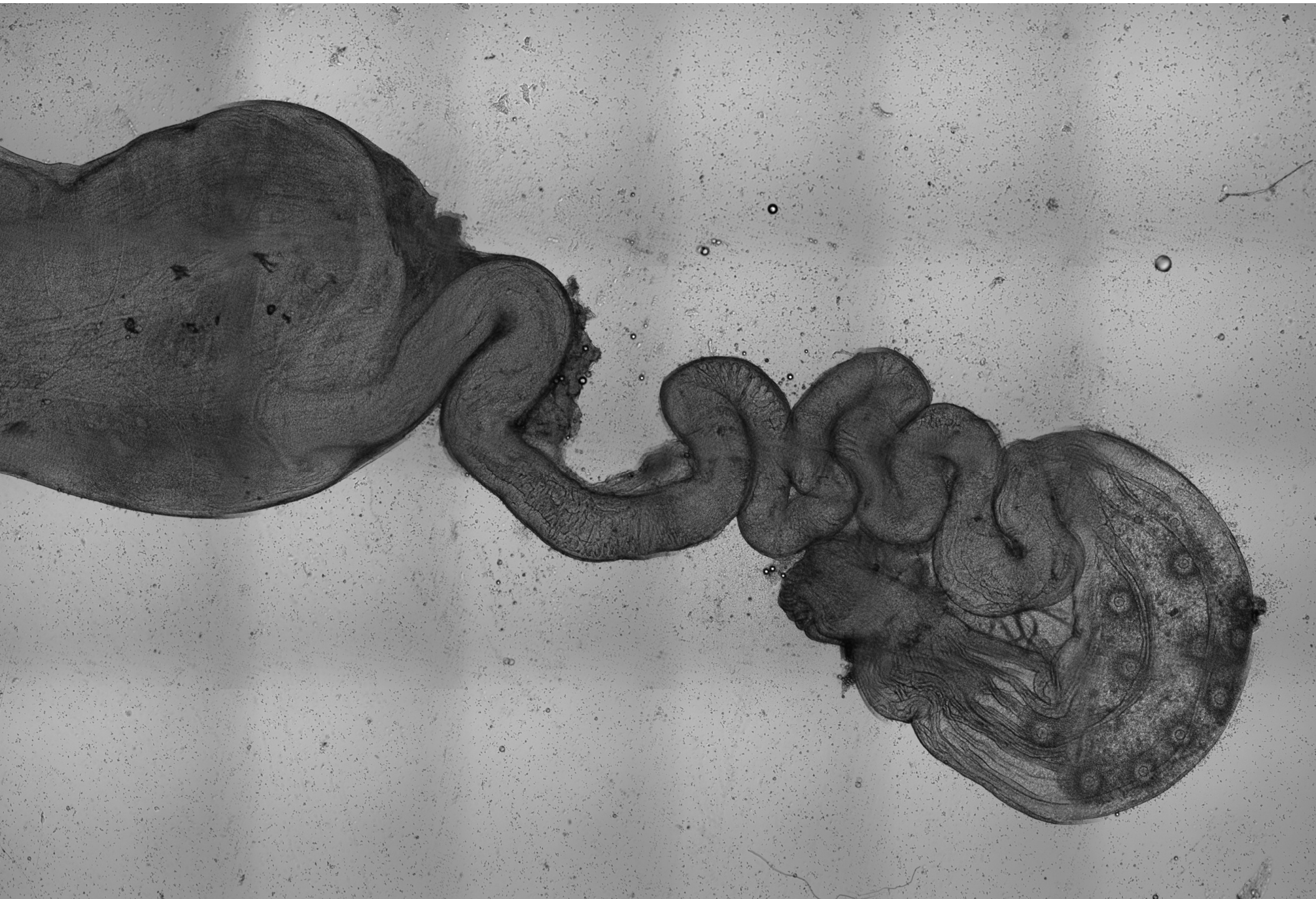

Supplement: Figure 1—source data 3. [file elife-97105-fig1-data3.pdf]

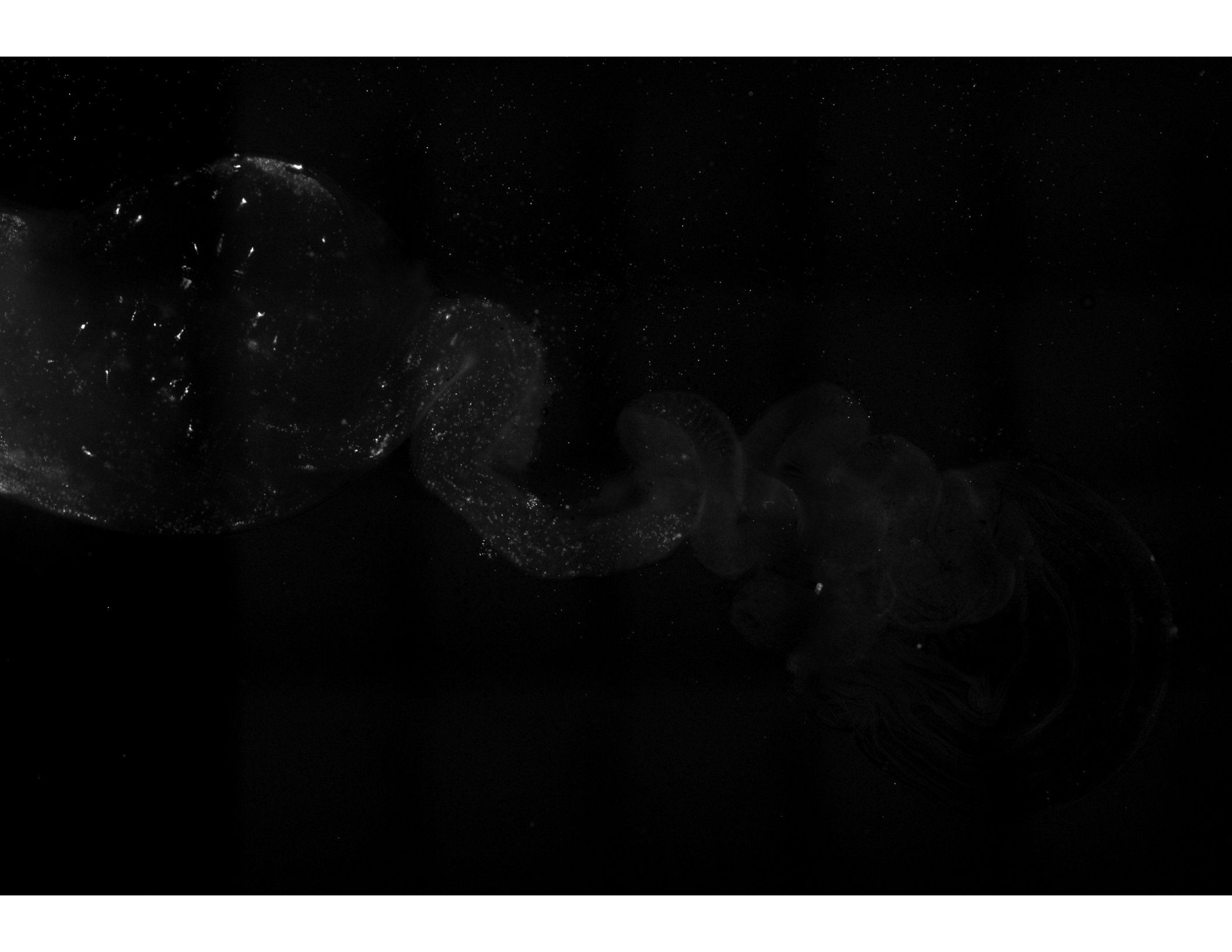

Supplement: Figure 1—source data 4. [file elife-97105-fig1-data4.pdf]
