# Supplementary material for: The epididymis contributes to sperm DNA integrity and early embryo development through Cysteine-Rich Secretory Proteins
Source: eLife. 2025 Apr 28;13:RP97105. doi: 10.7554/eLife.97105 (PMC12037180; doi:10.7554/eLife.97105)
Supplement: Supplementary file 1. [file elife-97105-supp1.docx]

*Supp Table 1. Analysis of different parameters in fresh and capacitated sperm*

|  | Control | | C1/C3 DKO | |
| --- | --- | --- | --- | --- |
|  | Fresh | Capacitated | Fresh | Capacitated |
| Sperm count (10^6^/ml) | 48,35 ± 3,83 | --- | 59,87 ± 12,03 | --- |
| Viability (%) | 66,22 ± 5,25 | 61,04 ± 2,78 | 65,54 ± 2,29 | 58,21 ± 4,31 |
| Progressive motility (%) | 63,99 ± 1,14 | 57,91 ± 2,39 | 62,65 ± 2,37 | 54,27 ± 3,19 |

Note: The percentages of sperm viability and progressive motility were calculated as the mean of at least 5 independent experiments.
